# A rare case of septic arthritis of the knee caused by *Salmonella typhi* with preexisting typhoid fever in a healthy, immunocompetent child – A case report

**DOI:** 10.1016/j.ijscr.2020.12.003

**Published:** 2020-12-03

**Authors:** Aryadi Kurniawan, Immanuel Panca Sitorus, Tonny Loho, Witantra Dhamar Hutami

**Affiliations:** a Pediatric Orthopaedic Surgeon, Department of Orthopaedic & Traumatology, Cipto Mangunkusumo National Central Hospital and Faculty of Medicine, Universitas Indonesia, Jalan Diponegoro No. 71, Jakarta Pusat, Jakarta 10430, Indonesia; b Department of Orthopaedic and Traumatology, Department of Orthopaedic & Traumatology, Cipto Mangunkusumo National Central Hospital and Faculty of Medicine, Universitas Indonesia, Jalan Diponegoro No. 71, Jakarta Pusat, Jakarta 10430, Indonesia; c Department of Clinical Pathology, Cipto Mangunkusumo National Central Hospital and Faculty of Medicine, Universitas Indonesia, Jalan Diponegoro No. 71, Jakarta Pusat, Jakarta 10430, Indonesia

**Keywords:** Septic arthritis, Typhoid fever, *Salmonella typhi*, Case report

## Abstract

**Introduction:**

Septic arthritis is a rapid and progressive infection caused by invasion of bacteria into the synovial joint. Disease of the joint causedby Salmonella spp in healthy children is an unusual event, with an estimated incidence of 0.1 to 0.2% of septic arthritis cases among children. The incidence of knee septic arthritis caused by *Salmonella typhi* with preexisting typhoid fever is very rare.

**Method:**

We reported a case of 2-years old boy with a history of saddle-type fever 2 weeks prior to right knee pain. Typhoid fever was confirmed by immunoassay test. Knee septic arthritis was established from clinical findings, increased CRP level, ultrasonography, and joint aspiration. Culture of the aspirate subsequently grew *Salmonella typhi*. This case report had been reported in line with SCARE criteria.

**Result:**

Arthrotomy and debridement were immediately performed.Intravenous piperacillin tazobactam was given for 6 days and replaced by amoxicillin clavulanic acid after the culture and sensitivity test was available. Patient recovered completely 5 months post surgery and showed excellence result with normal range of knee joint motion.

**Conclusion:**

This case report suggests that any episode of joint swelling following preexisting typhoid fever should arise the physician’s awareness toward the possibility of septic arthritis and warrant immediate as well as proper management.

## 1 Introduction

Septic arthritis accounts for approximately 0.25% of hospitalizations among children and majority of cases were identified in children younger than 2 years [1]. There were around 350 cases of septic arthritis per year in age group below 2 years old [1,2]. Etiologic organism varies by age and *S. aureus* is the most common causative pathogen identified in all age groups followed by group A *streptococcus, Haemophilus influenzae type b*, *Streptococcus pneumoniae*, and *Brucella melitensis* [3]. Localized salmonella infections usually present after Salmonella bacteremia, but sometimes it can occur after enteric fever or gastroenteritis. The dissemination of infection can occur, however, septic arthritis due to salmonella infections especially in knee joint is a rare entity. Based on the literatures, most of the patients affected by such disease have underlying chronic disease of immunosuppressive state [4]. Septic arthritis caused by Salmonella spp in healthy children is an unusual event, with an estimated incidence of 0.1–0.2% of septic arthritis cases among children [5].

This case report describes a rare case of a septic arthritis of knee joint caused by Salmonella in a child with preexisting typhoid fever. The aim of this study is to describe the occurrence of septic arthritis by *Salmonella typhi* in healthy, immunocompetent child therefore care must be taken to treat such kind of condition in the future. This case report had been reported in line with SCARE criteria [6].

## 2 Patient Information

We presented a 2-year-old male toddler who suffered from an episode of intermittent fever. The paeditrician confirmed that he had typhoid fever and treated the child accordingly. After one week of treatment, the fever subsided.

One week after the fever subsided, patient had right knee pain and limping while walking. He had to bend his right knee to relieve the pain. The knee was swollen and it got worse with time. He was brought to a bone-setter 4 times to have massages but the swelling got even worse and the fever arouse again. Patient eventually was admitted to Cipto Mangunkusumo General Hospital. Patient had no history of diseases causing immune-compromise in his body. Other than medication for previous thypoid fever, patient had no history of medication use.

## 3 Clinical findings

**Fig. 1:**
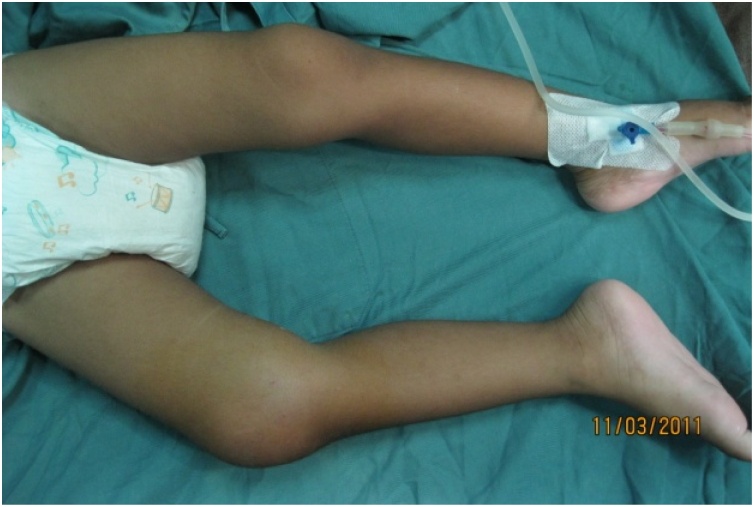
**Clinical Feature of the Right Knee Joint**. There is significant swelling on the right knee with flexion deformity and restricted movement.

Local state showed swollen knee (circumferential of 27 cm on right knee and 21 cm on contralateral knee) with tenderness, increased local temperature, flexion deformity and restricted movement of the right knee (Fig. 1).

## 4 Timeline

**Table:** 

**Time**	**Clinical Finding**	**Treatment**
**Two weeks before joint symptoms**	Intermittent fever	Treatment for typhoid fever with the duration of one week was performed by Pediatrician
**One week after fever subsided**	Pain and swollen right knee with limping gait	Piperazillin tazobactam after joint fluid was aspirated
**Within 24 h after result of joint fluid analysis was released**	Pain and swollen right knee with limping gait. Laboratory findings showed signs of infection	Arthrotomy and debridement

## 5 Diagnostic assessment

Laboratory tests results showed decreased haemoglobin (10.1 g/dL), increased erythrocyte sedimentation rate (93 mm), C-reactive protein (27 mg/L) and lactate dehydrogenase (509 IU/L). Peripheral blood smear showed microcytic hypochromic anaemia. IgM anti Salmonella showed positive result (8.0).

**Fig. 2:**
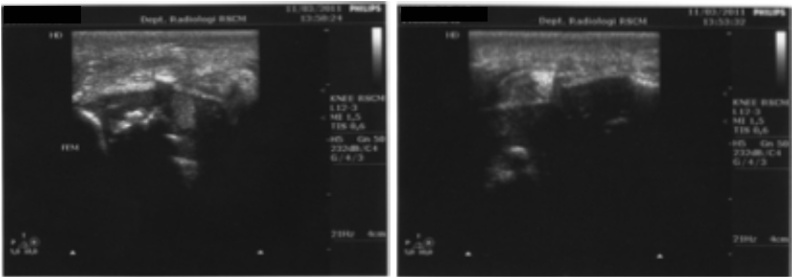
**Ultrasound Examination of the Right Knee.** The finding was marked joint effusion.

**Fig. 3:**
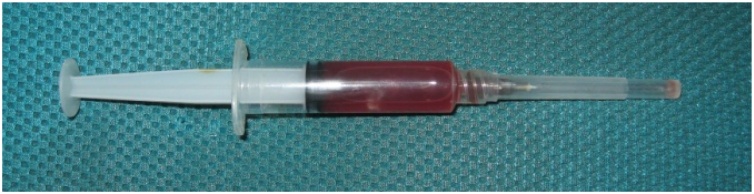
Joint Aspiration with Yellowish, Serohemorrhagic Fluid.

Ultrasound was performed and it showed a marked joint effusion with hyperechoic shadow. Needle aspiration was conducted under sonographic guidance (Fig. 2) and it yielded yellowish, serohaemorrhargic fluid. Gram stained smear of the joint fluid revealed plenty of leucocytes. Cytologic analysis of fluid aspiration showed signs of infection with turbid reddish fluid, decreased viscosity, poor mucin cloot test macroscopically, and increased cell count (13,200/μL) with polymorphonuclear predominant (90%) (Fig. 3).

**Fig. 4:**
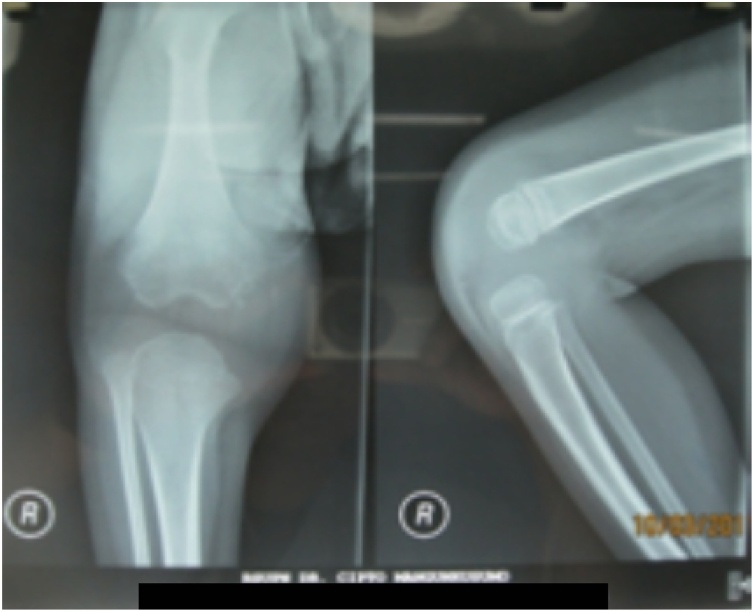
**Plain Radiography of the Right Knee Joint. The findings were** irregularity of the epiphysis with widening of joint space and soft tissue swelling.

Plain radiograph showed joint space widening on the right knee with soft tissue thickening and also epiphyseal irregularity of distal femur (Fig. 4).

## 6 Therapeutic intervention

Arthrotomy and debridement were performed immediately by the pediatric orthopaedic surgeon in our center who has more than a decade of experience (AK). Simultaneously intravenous piperacillin tazobactam was administered. Intraoperatively, seropurulen fluid was yielded when the joint capsule was opened, and both speciment of joint capsule and pus were taken for bacteria isolation. The administration of intravenous antibiotic was tolerated by the patient, and its administration was according to the prescribed regiment.

The isolate was identified as *S. typhi* by standard biochemical tests (VITEK 2 instrument from BioMeriux). Antibiotic susceptibility testing was done and was interpreted as per Clinical Laboratory Standards Institute Guidelines. The isolate was sensitive to Chloramphenicol, Cotrimoxazole, Gentamycin, Tetracycline, Amikacin, Sulbactam/Ampicillin, Cefotaxime, Amoxicillin clavulanic acid, Ceftriazone, Ceftazidime, and Meropenem. Intravenous piperacillin tazobactam was then replaced with intravenous Amoxicillin clavulanic acid for 7 days.

## 7 Follow up and outcomes

After antibiotic regiment for 7 days, the patient recovered completely, as proven by clinical and laboratory findings (decreased of CRP value to 15 mg/L on the 5th day). The patient then was discharged with oral amoxicillin clavulanic acid to be continued for 7 days.

**Fig. 5:**
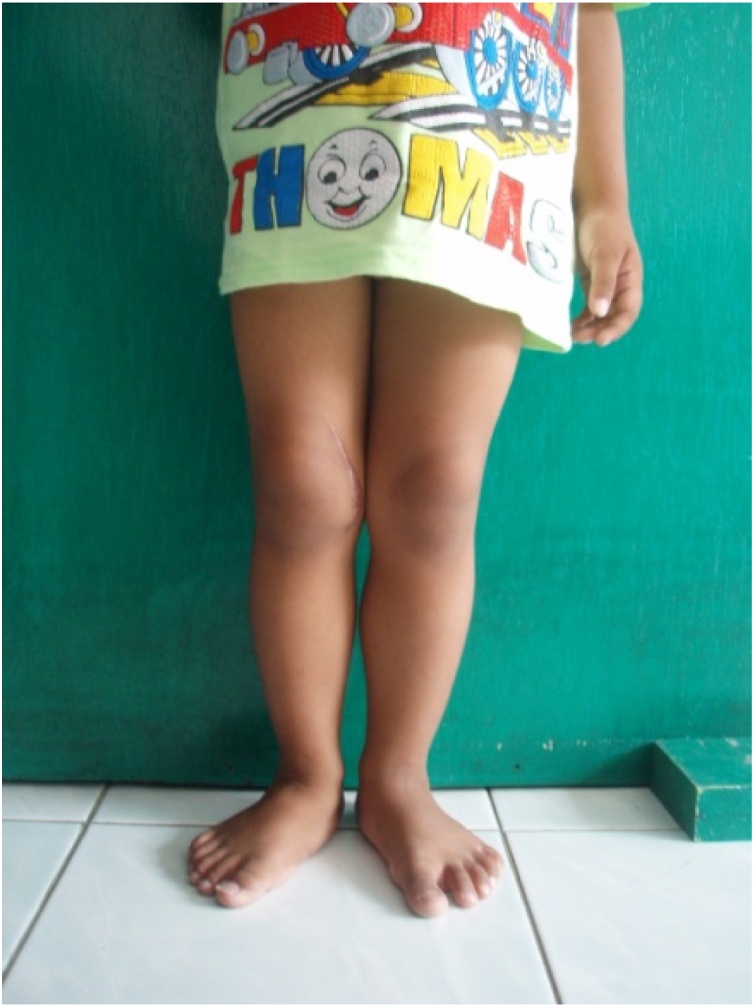
**Clinical Feature (5 Months Post Surgery).** There is no swelling and normal range of movement of right knee joint.

Patient had excellent knee joint motion at the fifth-month without disturbance on his right knee while standing, walking, or running (Fig. 5).

## 8 Discussion

The incidence of of septic arthritis in children was 5.5–12 cases per 100.000 individuals with predominant single joint infection and most common site at knee joint (40–55%) [5,6]. Early diagnosis of septic arthritis in children is very important because delayed or inadequate treatment carries a risk of permanent disability. Diagnosis of typhoid fever in our patient was established from the presence of intermittent fever, gastroenteritis symptoms, signs of acute inflammation with increased WBC count, and positive immunoassay test. Meanwhile, knee septic arthritis was diagnosed based on the pain with swollen knee joint, limping with limitation of joint motion, increased ESR and CRP level, plain radiograph, ultrasonography, and synovial joint aspiration. Acute septic arthritis is likely to occur in children younger than 5 years with a male preponderance which is appropriate with the patients’s condition [[7], [8], [9]].

Pathogen identification from synovial fluid established the diagnosis. All results established the diagnosis of knee septic arthritis caused by *Salmonella typhi*. These diagnosis criterias is appropriate to the Boston Children Hospital Clinical Practical Guideline [10].

In most septic arthritis, *Salmonella* is not suspected as common etiology and the diagnosis is established following its isolation. However, 10–20% of clinically diagnosed bacterial arthritis cases are never confirmed by positive synovial fluid or blood culture, especially in septic arthritis of the knee joint [7,13].

Septic arthritis caused by *Salmonella* is very rare, occurs in approximate 1% of all cases [11]. Usually it presents as one of the spreading infections in children with non-typhoidal *Salmonella* bacteremia following earlier episodes of gastroenteritis, as seen in this patient [11]. Similar to this case, most salmonella septic arthritis are monoarticular and the knee is the second most common joint affected after the hip joint. The synovium is a particular metastatic focus of salmonella infection [12].

Salmonella infection was suspected as the etiology of septic arthritis in this patient because of positive result (8.0) of *Salmonella* immunology test (IgM) [10]. Gram-staining of synovial fluid is positive in about half of the cases and faecal culture is positive in 43%. Isolation of *Salmonella typhi* from the synovial fluid at the knee joint was succeeded in this patient and this finding further established the diagnosis. The blood and fecal culture were not conducted in this patient.

Salmonella septic arthritis is more likely occur in patients with preexisting disease such as hemoglobinopathy especially sickle cell disease in children, prior joint disease (rheumatoid arthritis), hematologic neoplasm, SLE, administration of corticosteroid or other immunosuppressant treatment, idiopathic trombocytopenia, alcoholic liver disease, AIDS, and disease that produce an increased haemolysis. Typhoid fever as a preexisting disease of *Salmonella* septic arthritis was a rare condition. These preexisting diseases can contribute to chronic carrier state of *Salmonella* infection with higher incidence in developing nations [13]. Our patient had only mild hypochromic microcyctic anemia. Routine blood test in this patient indicated an acute inflammation.

Along with the preexisting disease, the inadequate treatment prescribed by a physician for fever may have resulted in prolonged exposure to the organism. Hematogenous inoculation of the pathogenic organism is usually followed with infectious arthritis. It has been reported that several children have developed Salmonella reactive arthritis in about 2 weeks after initial diarrheal episode and enteric fever [10] and this condition is similar to this cases in which the patient experienced diarrhoea and fever 2 weeks before the knee pain.

Definitive therapy for septic arthritis is based on the identification and antibiotic susceptibility of the bacteria isolated in culture. Most enteric Gram-negative infections can be treated in 2–4 weeks by second or third generation cephalosporins given intravenously or fluoroquinolone. Intraarticular antibiotic instilation is contraindicated since they may induce chemical synovitis [10] Our patient responded well to medical and surgical treatment. Intravenous Piptazobactam as empirical antibiotic was given for 6 days, then replaced with Amoxycillin clavulanic acid for 1 week after the result of bacteriologic culture and sensitivity. Surgical decompression for septic arthritis in young children is the best management because arthrotomy can threaten the cartilage. Patient can do daily activities without limitation and painon the right knee 5 months after the surgery. These outcomes demonstrated that the patient had good response to the treatment.

## 9 Conclusion

In summary, Salmonella septic arthritis of the knee is a very rare manifestation of salmonella infection and it can turned into chronic carrier state when there are preexisting disease and compromised host defence. This case is reported to highlight the unusual presentation of *S. Typhi*. Every case with clinical diagnosis of septic arthritis should be properly investigated, and joint fluid with blood culture should always be performed since bacteremia is a constant feature of enteric fever and its dissemination may lead to localized foci of infection including bones and joints. Antibiotic treatment and surgical decompression should be initiated by the time the clinical diagnosis is made. Timely intervention and correct diagnosis and treatment in this child’s case have saved the affected knee joint, which otherwise would have been permanently damaged for his lifetime.

What we can add to the literature is that septic arthritis caused by *Salmonella typhi,* despite rare, can occur to healthy, immunocompetent patient due to hematogenous spread from systemic Salmonella infection. One must consider this etiology when facing patient, particularly pediatric patient, with joint symptoms and history of recent systemic infection with whatever the causative pathogen.

## Patient perspective

Patient’s parents had been informed and acknowledged the disease course and therapeutic planning and outcomes for the patient.

## Declaration of Competing Interest

The authors certify that they have NO affiliations with or involvement in any organization or entity with any financial interest or non-financial interest in the subject matter or materials discussed in this manuscript.

## Funding

The authors received no financial support for the research, authorship, and/or publication of this article.

## Ethical approval

The ethical approval was not required for this case report.

## Consent

Written informed consent was obtained from the patient for publication of this case report and accompanying images. A copy of the written consent is available for review by the Editor-in-Chief of this journal on request.

## Author contribution

Aryadi Kurniawan: study concept, data collection, data interpretation, and writing the paper

Immanuel Panca Sitorus: data collection, data interpretation and writing the paper

Tonny Loho: data collection, data interpretation and writing the paper

Witantra Dhamar Hutami: data collection, data interpretation and writing the paper

## Registration of research studies

Not applicable.

## Guarantor

Aryadi Kurniawan.

## Provenance and peer review

Not commissioned, externally peer-reviewed.
